# Impact of Telehealth on Health Disparities Associated With Travel Time to Hospital for Patients With Recurrent Admissions: 4-Year Panel Data Analysis

**DOI:** 10.2196/63661

**Published:** 2024-11-25

**Authors:** Youyou Tao, Ace Vo, Dezhi Wu, Junyuan Lin, Kala Seal, Abhay Mishra, Arindam Brahma

**Affiliations:** 1 Loyola Marymount University Los Angeles, CA United States; 2 University of South Carolina Columbia, SC United States; 3 Iowa State University Ames, IA United States

**Keywords:** telehealth, health disparities, travel time, length of stay, recurrent admissions, health care access, virtualization potential, virtual care

## Abstract

**Background:**

Geographic, demographic, and socioeconomic differences in health outcomes persist despite the global focus on these issues by health organizations. Barriers to accessing care contribute significantly to these health disparities. Among these barriers, those related to travel time—the time required for patients to travel from their residences to health facilities—remain understudied compared with others.

**Objective:**

This study aimed to explore the impact of telehealth in addressing health disparities associated with travel time to hospitals for patients with recurrent hospital admissions. It specifically examined the role of telehealth in reducing in-hospital length of stay (LOS) for patients living farther from the hospital.

**Methods:**

We sourced the data from 4 datasets, and our final effective sample consisted of 1,600,699 admissions from 536,182 patients from 63 hospitals in New York and Florida in the United States from 2012 to 2015. We applied fixed-effect models to examine the direct effects and the interaction between telehealth and patients’ travel time to hospitals on LOS. We further conducted a series of robustness checks to validate our main models and performed post hoc analyses to explore the different effects of telehealth across various patient groups.

**Results:**

Our summary statistics show that, on average, 22.08% (353,396/1,600,699) of patients were admitted to a hospital with telehealth adopted, with an average LOS of 5.57 (SD 5.06) days and an average travel time of about 16.89 (SD 13.32) minutes. We found that telehealth adoption is associated with a reduced LOS (*P*<.001) and this effect is especially pronounced as the patients’ drive time to the hospital increases. Specifically, the coefficient for drive time is –0.0079 (*P*<.001), indicating that for every additional minute of driving time, there is a decrease of 0.0079 days (approximately 11 minutes) in the expected LOS. We also found that telehealth adoption has a larger impact on patients frequently needing health services, patients living in high internet coverage areas, and patients who have high virtualization potential diseases.

**Conclusions:**

Our findings suggest that telehealth adoption can mitigate certain health disparities for patients living farther from hospitals. This study provides key insights for health care practitioners and policy makers on telehealth’s role in addressing distance-related disparities and planning health care resources. It also has practical implications for hospitals in resource-limited countries that are in the early stages of implementing telehealth.

## Introduction

The World Health Organization has stated that health services quality should not vary by factors such as ethnicity, gender, socioeconomic status, and geographic location [1]. Unfortunately, geographic, demographic, and socioeconomic differences exist in health outcomes despite the focus on such issues by health organizations worldwide [2,3]. Such disparities have adverse effects on particular groups, as evidenced by disproportionately worse medical outcomes [2,3]. The barriers to access care for these populations contribute to significant health disparities, defined as health differences related to economic, social, or environmental disadvantages [4]. Compared with other access barriers, access barriers related to travel time (ie, the time required for patients to travel from their residences to health facilities) remain understudied [5].

This paper focuses on this particular access barrier, travel time. Previous studies found that travel time to the hospital can cause considerable health disparity [6,7]. Recent literature has explored the effect of travel time on patients’ in-hospital length of stay (LOS)—the number of days a patient stays in the hospital from admission to discharge—and found mixed results. For example, Simpson et al [8] found no difference in LOS based on travel time, while Jackson et al [9] discovered a positive association between these 2 variables. Rana et al [10] reported that the mean difference for LOS among patients with chronic obstructive pulmonary disease traveling short (0-15 km) and intermediate (>15-50 km) distances was statistically insignificant. However, patients traveling long distances (>50 km) had a significantly shorter LOS compared with both short- and intermediate-distance patients. This study suggested that this could be due to the distance decay phenomenon, where the interaction between 2 locations diminishes as the distance between them increases [10]. Specifically, patients who live farther from the hospital might experience a shorter LOS compared with those residing closer [10]. This distance decay phenomenon is also supported by other studies, which have shown that compared with patients living closer to health care facilities, those living farther away had worse health outcomes such as lower survival rates in hospitals and higher rates of nonattendance at follow-up due to the longer distance [6,7].

While documenting the existence of health disparities that are associated with travel time to the hospital, scholars suggested that future studies should focus on examining how travel-related access barriers can cause adverse patient outcomes and further propose feasible interventions that could mitigate these health disparities [11]. Telehealth, defined as “the use of electronic information and telecommunication technologies to support long-distance clinical health care, patient and professional health-related education, health administration, and public health [12],” can help by providing virtual care that allows patients to manage their conditions and communicate with health care providers without traveling [13-15]. A 2022 report from the American Medical Association’s Physician Practice Benchmark Surveys shows that 74.4% of physicians reported using telehealth in their practice [16]. Telehealth is commonly used in specialties such as cardiology, emergency medicine, family medicine, hematology, general internal medicine, neurology, obstetrics and gynecology, oncology, and pediatrics [17]. However, even with the growing adoption of telehealth in clinical settings, the specific impact of telehealth on health outcomes related to travel time remains underexplored.

Among various health outcome measures, our study aims to investigate how telehealth can reduce disparities in in-hospital LOS. Reducing LOS can not only alleviate the financial burden on patients but also contribute to the broader impact of decreasing societal health care costs [18]. Various health information technology (HIT) interventions, including telehealth, have been shown in the literature to statistically significantly reduce LOS [18-20]. Telehealth, in particular, has been shown to improve health outcomes, including LOS reduction, through two primary mechanisms: (1) real-time interventions that require real-time interaction between patients and their health providers, such as video conferencing, telephone communication, and telemonitoring equipment, facilitating immediate patient-provider interaction and timely care management [21-23], and (2) asynchronous interventions that do not require immediate interaction between patients and their health providers, including remote monitoring systems, interactive platforms, and in-home secure messaging through a patient portal using either a phone or a computer, which provide access to patient educational resources and enable continuous patient monitoring, with patients’ data reviewed at a later time for medical treatment [15,24-26]. Through these mechanisms, telehealth, alongside other HIT applications, plays a significant role in shortening LOS by enabling early intervention through the efficient gathering of useful information [27] and facilitating consultation, diagnosis, treatment, care management, education, and patients’ self-care [28]. Studies have shown that telehealth decreased LOS for patients in progressive care units without significant cost increases [29], for patients with vigorous chronic obstructive pulmonary disease after 7 months of monitoring [27], and for patients in rural and small hospitals through real-time telehealth interventions [30]. However, to the best of our knowledge, no study has specifically explored how telehealth could mitigate health disparities associated with travel time to hospitals in terms of LOS.

Given the pressing need to address health disparities associated with travel time to hospitals, our study aims to comprehensively examine how health disparity in patients’ in-hospital LOS caused by long travel time to the hospital could be mitigated through telehealth implementation [27,29,30]. Our overarching research question is, “How can telehealth adopted by hospitals mitigate the adverse effects of travel time to the hospital, reduce health disparity, and influence patients’ health outcomes in terms of LOS?”

## Methods

We present the *Methods* section following the STROBE (Strengthening the Reporting of Observational Studies in Epidemiology) guidelines, and we also provided a checklist of items for the STROBE statement in Multimedia Appendix 1 [31].

### Study Design and Study Setting

The data applied in this study were sourced from 4 sources. First, we used data from the Healthcare Cost and Utilization Project’s State Inpatient Databases (HCUP-SID) from 2012 to 2015 to obtain patient characteristics, admission, and clinical information [32]. The patient’s zip code information was part of this dataset. Second, we applied data from the American Hospital Association (AHA) annual surveys from 2012 to 2015 to obtain hospital characteristic data [33] including hospital location information such as latitude and longitude. Using this locational data and patient zip code, we computed “drive time to hospital” for each patient. Third, we used AHA’s information technology (IT) supplement files for 2011-2014 to obtain HIT implementation data [34]. Considering the lagged effects of HIT adoption, we followed the method that has been widely adopted to map the datasets with HIT variables lagged by 1 year [35]. Fourth, we collected additional census-based data, such as English proficiency level at the census tract level. We then matched the reference data between the census tracts and the United States Postal Service’s zip codes to allocate each census tract data proportionally.

**Figure 1:**
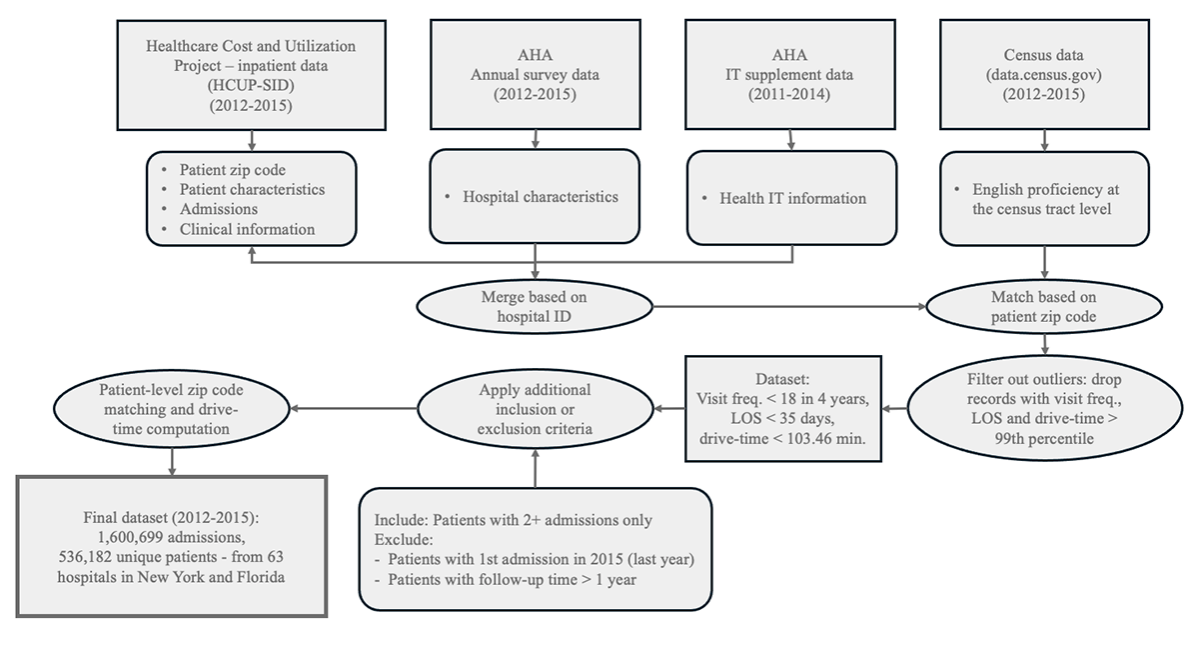
Data preparation method. AHA: American Hospital Association; freq.: frequency; HCUP-SID: Healthcare Cost and Utilization Project’s State Inpatient Datasets; IT: information technology; LOS: length of stay.

We merged data from the Healthcare Cost and Utilization Project (HCUP), AHA surveys, and AHA IT files with unique hospital identifications before merging the datasets with census data using patients’ zip codes from HCUP-SID. Furthermore, we filtered out data points beyond the 99th percentile for LOS, patient visit frequency, and travel time. To remove the outliers, we only included patients with an in-hospital stay of fewer than 35 days, fewer than 18 hospital visits over 4 years, and a travel time of less than 103.46 minutes in our sample data. To examine the effect of telehealth on recurrent hospital admissions, we only kept patients with 2 or more admissions in our sample. To ensure a consistent follow-up period for each patient, we excluded those whose first admission occurred in the dataset’s final year (2015), applying a 1-year cutoff for the follow-up duration. Our final effective sample consists of 1,600,699 admissions originating from 536,182 patients within 63 hospitals in New York and Florida in the United States from 2012 to 2015. This final dataset contains no missing values. Our data processing procedure is visualized in Figure 1. We chose datasets from New York and Florida because the high volume of hospital admissions in these 2 states and the availability of linkage variables that allowed us to connect patients with their hospital admissions so that we can examine telehealth’s impact on patients with recurrent admissions.

### Study Outcome

We applied in-hospital LOS to investigate telehealth’s impact on patients’ health outcomes [20,36]. In-hospital LOS, measured as the number of days of hospital stays from admission to discharge, serves as a proxy for hospital operations efficiency measures [20,36].

### Study Variables

Our main study variables were telehealth implementation and travel time to the hospital. We treated telehealth implementation in hospitals as a categorical variable, where 1 indicates telehealth is adopted by the hospital—meaning the hospital has a computerized system that supports telehealth—and 0 otherwise [33]. Telehealth implementation measured by this item includes health care services provided at a distance through various modalities such as remote monitoring, video conferencing, wireless communications, and electronic consults [37].

Travel time to the hospital was calculated using the ArcGIS Network Analyst (Environmental Systems Research Institute) extension with the origin-destination cost matrix. The matrix provides both travel time and travel distance along the shortest route from the origins—patients’ locations, represented by the centroids of their zip codes—to the destinations, that is, hospitals’ physical addresses. To obtain travel time and travel distance, the origin-destination cost matrix was solved within ArcGIS Pro version 3.3.1 using the native Network Analyst solver. This solver is a subscription service from Environmental Systems Research Institute, which maintains and regularly updates the network solver. The process of obtaining drive time is as follows:

1. Geocode (transforming text addressed to geospatial data point) the hospital addresses as points on the map, retaining hospital ID as identifiers
2. Obtain New York and Florida zip code shapefiles from the census (which are equivalent to the United States Postal Service’s zip code)
3. Convert the zip code shapefiles, which are polygons (spatial areas), to their respective centroids (midpoints of the polygons) as points on the map, retaining zip codes as identifiers
4. Link patients’ zip codes to the respective points on the map. The points will serve as proxies of the actual patients’ addresses
5. For each state (New York and Florida), assign the unique patients’ zip code locations as origins and hospital addresses as destinations, then calculate the origin-destination cost matrix to obtain pair-wise travel time and travel distance for each origin and destination
6. Link the travel time and travel distance from the origin-destination cost matrix to the respective encounter data using patient zip codes and hospital IDs

We also included community-level, hospital-level, patient-level, and admission-level variables as control variables to account for other factors that may influence in-hospital LOS. At the community level, we included the English proficiency level, measured by the percentage of the population that speaks English less than well in each zip code. Studies showed that compared with patients without a language barrier, patients with a language barrier experience more health care disparity in care, including longer LOS [38]. For patients to use telehealth services from home, the first requirement is to have internet access, and recent studies show that internet access can empower patients, influence health outcomes, and change their health service usage behaviors [39,40]. Thus, we also controlled the percentage of internet coverage at the zip code level.

We applied hospital characteristics at the hospital level that may influence in-hospital LOS, such as hospital bed size, profit status, in a health system or not, and market share. Market share is measured by the Herfindahl-Hirschman Index. We also included HIT variables to control for the other HIT effects on LOS, which are clinical decision support system, computerized provider order entry, electronic clinical documentation, results viewing, and health information exchange (refer to Multimedia Appendix 2 for the detailed list of technologies and coding for HIT control variables).

At the patient level, we included age at discharge, gender, and race. At the admission level, we included insurance types, including Medicare, Medicaid, and private insurance; the total number of comorbidities; chronic diagnoses; diagnoses; procedure; total charge at the current admission; the number of times the patient visited the hospitals since the first hospital visit; and the number of days between the current visit and the previous visit. We used 17 different types of body systems (refer to “Disease types” in Multimedia Appendix 3 for details) and a dummy variable that records whether the patient’s primary diagnosis is in the same body system at the current admission compared with the last admission. We presented the summary statistics of control variables and additional variables for post hoc analysis in Multimedia Appendix 3.

### Ethical Considerations

The HCUP data used in this study are classified as a limited dataset, as defined by the HCUP website [41]. According to the website [41], “HCUP databases are limited data sets. HCUP databases conform to the definition of a limited data set. A limited data set is healthcare data in which 16 direct identifiers, specified in the Privacy Rule, have been removed. Under HIPAA, review by an institutional review board is not required for use of limited data sets.” The AHA’s annual surveys and IT supplement files used in this study were collected at the hospital level and did not involve human participants. This study was granted exemption from institutional review board review by Loyola Marymount University’s Institutional Review Board (LMU IRB 2024 FA12-R).

### Statistical Analysis

After conducting descriptive analyses, we used STATA/SE 18.0 (StataCorp) for the subsequent data analysis. In this study, fixed-effect regression models were applied to assess the impact of telehealth on in-hospital LOS [42]. The model is specified in equations 1 and 2.

LOS_ijtv_ = β_0_ + β_1_Telehealth_j,t–1_ + β_2_Drive_Time_ijtv_ + Control_ijtv_η + λ_t_ + α_v_ + γ_j_ + ε_itv_ (1)

In equation 1, *LOS_ijtv_* is the dependent variable that represents the in-hospital LOS for patient *i*’s *v*th hospital visit in hospital *j* at year *t*. *Telehealth_j,t–1_* indicates whether telehealth was adopted by hospital *j* in the previous year (*t−1*). *Drive_Time_ijtv_* represents the drive time for patient *i*’s *v*th hospital visit to hospital *j* in year *t*. The focal parameters of interest in this model are *β_1_* and *β_2_*, which capture the direct effect of telehealth implementation by hospital *j* in the previous year (*t–1*) and the direct effect of drive time for patient *i* to hospital *j* for the *v*th visit at year *t. Control_ijtv_* represents community-level, hospital-level, patient-level, and admission-level factors as described above that may influence patient in-hospital LOS. We include *γ_j_* in the model to account for the hospital fixed effects that absorb unobserved hospital characteristics that do not vary over time, *λ_t_* to absorb the change over time captured by the year fixed effect, *α_v_* to account for the fixed effects for hospital visit number that absorb the unobserved factors that could vary with each hospital visit, and *ε_itv_* as the idiosyncratic error term.

LOS_ijtv_ = β_0_ + β_1_Telehealth_j,t–1_ + β_2_Drive_Time_ijtv_ + β_3_Telehealth_j,t–1_ × Drive_Time_ijtv_ + Control_ijtv_η + λ_t_ + α_v_ + γ_j_ + ε_itv_ (2)

In equation 2, the focal parameter of interest is *β_3_*, which captures the interaction effect between telehealth and drive time to capture how the effect of telehealth on LOS varies with patients’ drive time to the hospital. In both models, robust SEs were applied to account for potential heteroskedasticity, and the errors were further clustered at the patient level to account for the possible within-patient correlations.

## Results

### Impact of Telehealth and Travel Time

Table 1 presents summary statistics of our main variables. Our summary statistics show that, on average, 22.08% (353,396/1,600,699) of patients were admitted to a hospital with telehealth adopted, the average LOS was 5.57 (SD 5.06) days, and the average travel time was about 16.89 (SD 13.32) minutes.

**Table 1:** Summary statistics for main variables.

Variable name	Variable, mean (SD)	Variable, minimum	Variable, maximum
Telehealth^a^	0.22 (0.42)	0	1
Length of stay (in days)	5.569 (5.06)	0	34
Travel time (in minutes)	16.89 (13.32)	0.89	103.43

^a^Dummy variables with 2 values (ie, 0 and 1).

**Figure 2:**
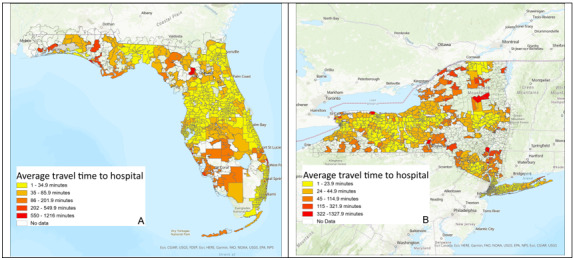
Travel time to hospital in minutes: (A) Florida and (B) New York.

To explore whether telehealth mitigates the effect of travel time to the hospital on patient in-hospital LOS, we first mapped the driving time to the hospital in minutes in Florida and New York in Figures 2A and 2B, respectively. From Figure 2, we observed that people with longer drive time to hospitals were distributed in both the north and south of Florida and on the edges of New York.

Next, we present our analysis result in Table 2. In Table 2, model 1, we can infer that both the direct effects of the implementation of telehealth and drive time are associated with a decreased LOS at the *P*<.001 level. Specifically, patients admitted to hospitals that have adopted telehealth services experience a reduction in in-hospital LOS by 0.0431 days (approximately 62 min) compared with those admitted to hospitals without telehealth implementation. Given the average hospital-adjusted expense per inpatient per day was US $3025 in the United States in 2022 [43], telehealth implementation can save around US $130.38 in hospital cost per patient stay.

**Table 2:** Effect of telehealth adoption and drive time to hospital on length of stay^a,b^.

Models			Main models				Robustness check										
			Main effect	Interaction effect added		Comorbidities added			Dynamic model		Patient-level FE^c^ model		NB^d^ model		Log (LOS^e^)		PSM^f^
			Model 1	Model 2		Model 3			Model 4		Model 5		Model 6		Model 7		Model 8
**Telehealth**																	
	Coefficient	–*0.0431*^*g*^		0.0056	0.0103			0.0035		0.0092		0.0009		0.0056		0.0310	
	*P* value	*<.001*		.71	.50			.85		.65		.73		.01		.25	
	Robust SE	*0.01*		0.02	0.02			0.02		0.02		0.003		0.002		0.03	
**Drive time**																	
	Coefficient	–*0.0079*		–0.0073	–0.0067			–0.0060		–0.0065		–0.0009		–0.0008		–0.0057	
	*P* value	*<.001*		<.001	<.001			<.001		<.001		<.001		<.001		<.001	
	Robust SE	*0.000*		0.000	0.000			0.000		0.001		0.000		0.000		0.001	
**Telehealth×Drive time**																	
	Coefficient	—^h^		–*0.0026*	–*0.0026*			–*0.0029*		–*0.0023*		–*0.0003*		–*0.0003*		–*0.0023*	
	*P* value	—		*<.001*	*<.001*			*<.001*		*.01*		*<.001*		*<.001*		*.03*	
	Robust SE	—		*0.001*	*0.001*			*0.000*		*0.000*		*0.000*		*0.000*		*0.001*	
**LOS_*v*–1_^i^**																	
	Coefficient	—		—	—			0.0564		—		—		—		—	
	*P* value	—		—	—			<.001		—		—		—		—	
	Robust SE	—		—	—			0.001		—		—		—		—	
**Admission-level controls**			Yes	Yes		Yes			Yes		Yes		Yes		Yes		Yes
**Patient-level controls**			Yes	Yes		Yes			Yes		Yes		Yes		Yes		Yes
**Hospital-level controls**			Yes	Yes		Yes			Yes		Yes		Yes		Yes		Yes
**Community-level controls**			Yes	Yes		Yes			Yes		Yes		Yes		Yes		Yes
**Year and hospital fixed effects**			Yes	Yes		Yes			Yes		Yes		Yes		Yes		Yes
**Comorbidities**			No	No		Yes			No		No		No		No		No
**Observations, n**			1,600,699	1,600,699		1,600,699			1,070,901		1,600,699		1,600,699		1,600,699		542,089
**Patients, n**			536,182	536,182		536,182			462,234		536,182		536,182		536,182		260,139
**Adjusted *R*^2^**			0.582	0.582		0.588			0.594		0.569		—		0.486		0.581

^a^Robust SEs clustered by patients.

^b^Constant is included in each of the models.

^c^FE: fixed effect.

^d^NB: negative binomial.

^e^LOS: length of stay.

^f^PSM: propensity score matching.

^g^Italicized values represent [insert reason].

^h^Not applicable.

^i^LOS*_v–_*_1_: length of stay for patient’s *v–*1st visit to the hospital.

From Table 2, model 1, we also found that the coefficient for drive time is –0.0079, with a *P* value <.001, indicating that longer drive times to the hospital are significantly associated with a slight decrease in LOS. For every additional minute of driving time, there is a decrease of 0.0079 days (approximately 11 min) in the expected LOS. From Table 2, model 2, we found that the interaction effect of the telehealth implementation and the drive time is associated with a decreased LOS at the *P*<.001 level, suggesting that telehealth may be particularly effective at reducing LOS for patients who live farther away from the hospital.

**Figure 3:**
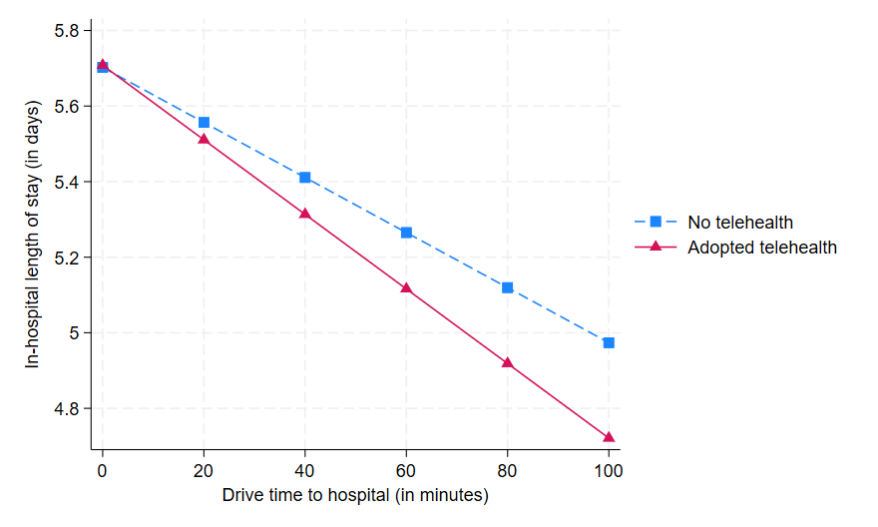
The impact of telehealth implementation on length of stay by drive time.

To visualize the impact of telehealth adoption on the LOS for patients with various travel times to the hospital, we plotted this effect in the interaction plot in Figure 3. We plotted 2 separate lines, 1 for hospitals that implemented telehealth and 1 for those that did not. We found that for patients admitted to hospitals with telehealth, there is a steeper decrease in the LOS as the drive time increases from 0 to 100 minutes (indicated by the solid red line with triangle markers). Conversely, for patients admitted to hospitals without telehealth, the LOS decreases as the drive time increases, but the decrease is not as steep (indicated by the dashed blue line with square markers). This result indicates telehealth implementation is more effective in reducing LOS for patients who live farther from the hospital.

To examine the robustness of our results, we conducted 5 sets of tests, the results of which are presented in Table 2, models 3-8. We first included an additional 29 types of comorbidities (refer to “Comorbidity type” in Multimedia Appendix 3 for more details) to control for the severity of the patient’s condition (result in model 3). Next, we incorporated the patients’ lagged LOS from the previous admission as an independent variable to account for the potential impact of past LOS on the current LOS (result in model 4). In our main models, we accounted for the hospital-level fixed effects but not the patient-level fixed effects. Accounting for hospital-level fixed effects allows us to absorb unobserved hospital characteristics that do not vary over time. However, by not including patient-level fixed effects, we may overlook unobserved patient characteristics that also do not vary over time. To address this in model 5, we considered patient-level fixed effects by introducing patient-specific intercepts in this model to control for unobserved, patient-specific characteristics that are time-invariant. We found the results from models 3-5 consistent with our main model.

Furthermore, in our main model, we applied LOS as our dependent variable for better result interpretation. According to the literature, despite the right-skewness of LOS, linear regression is validated as statistically sound for large data samples, even when the dependent variable is not normally distributed [44-46]. The variance of the LOS is 25.66 days, which exceeds the mean of 5.57 days, indicating overdispersion in the data. To further test the robustness of our results, we applied a negative binomial model to account for skewness and over-dispersion (result in model 6). Furthermore, we examined the impact of telehealth adoption and drive time to the hospital on the logarithmically transformed LOS (result in model 7). We found the results from both models 6 and 7 to be consistent with our main model.

In model 8, we used propensity score matching to construct a balanced comparison between the treatment group (ie, patients admitted to hospitals that have implemented telehealth) and the control group (ie, those admitted to hospitals without telehealth). We first calculated propensity scores using a logistic regression model with hospital-level factors (such as the implementation level of clinical decision support system, computerized provider order entry, electronic clinical documentation, results viewing, and health information exchange) and patient-level and admission-level (such as number of comorbidities, age, race, insurance type, current visit number, and body system categorizations). After matching patients using the k-nearest neighbors approach, we assessed the balance of hospital-level and patient-level variables between the treatment and control groups and found the standardized difference did not exceed a 10% threshold, indicating that treatment and control groups share similar statistical properties [47]. Postmatching analysis was conducted on the weighted and matched sample and clustered by patients to account for within-group correlations. The results, presented in model 8, are consistent with our main model. Overall, the results in models 3-8 show that our main results are robust and consistent among various robustness tests.

### Post Hoc Analysis

To explore the nuanced effects of the direct effect of telehealth adoption and the interaction effects between telehealth adoption and drive time to hospital for subgroups of patients, we conducted post hoc analyses and present the result in Table 3. We first explored whether telehealth adoption has a larger impact on patients who need frequent hospital visits and have a high level of comorbidity by dividing the patients into groups 1 and 2 based on their frequency of hospital visits and comorbidity counts. Furthermore, we investigated whether telehealth adoption has a larger impact on patients living in areas with high internet coverage compared with those in areas with low coverage by dividing the patients into groups 3 and 4 based on median internet coverage. The literature suggests that internet coverage plays a significant role in delivery of various types of health care and broadband access is indeed a social determinant of health [48]. Access to broadband-based internet can allow specialists to collaborate with doctors in rural areas for saving patients with stroke or improving access to mental health care for the veterans [49]. These are a few examples of the myriad ways that internet access, particularly access to broadband internet, can serve as an enabler of delivery of telehealth across the population. In fact, research further indicates that a lack of broadband access can create a barrier for accessing telehealth services [50]. For our research, we surmise that while driving time itself can be a determinant for the level of use of telehealth services, there is a prerequisite of the presence of broadband within the community for accessing and using the telehealth services. In other words, the internet coverage itself would play a moderating role in determining the access and effectiveness of telehealth services (as measured by LOS) and we are trying to capture that in our post hoc analyses.

**Table 3:** Post hoc patient group description.

Post hoc patient group	Visit frequency (median=2)	Inclusion condition	Comorbidity (median=3)	Internet coverage (median=80.04%)
Group 1: High frequency and high comorbidity	>2	AND	>3	—^a^
Group 2: Low frequency or low comorbidity	≤2	OR	≤3	—
Group 3: High internet coverage	—	—	—	≥80.04%
Group 4: Low internet coverage	—	—	—	<80.04%

^a^Not applicable.

In the subgroup analysis (Table 4), both the direct telehealth implementation effect and the interaction effects consistently reduce LOS for groups 1 and 2 (see the italicized results in Table 4), with a more pronounced effect for group 1, high frequency and high comorbidity group, indicating that telehealth could be effectively integrated into the care plans for patients who live farther from the hospitals, frequently engage with health care systems, and have complex health needs.

Furthermore, we found that telehealth decreases LOS for the high internet coverage group (group 3) but not in the low internet coverage group (group 4). We also found that the interaction effects between telehealth and drive time are consistently associated with reduced LOS for both groups, but the effect size is larger for patients living in high internet coverage areas (see the italicized results in Table 4). This highlights the importance of high internet coverage as a prerequisite to fully benefit from telehealth.

**Table 4:** Effect of telehealth adoption and drive time to hospital on length of stay by patient groups^a-d^.

Subgroup and effects				Telehealth	Drive time		Telehealth×Drive time		Observations, n	
**Group 1: High frequency and high comorbidity**										308,365
	**Direct effect**									
		Coefficient	–*0.0844*^e^		–0.0051	—^f^				
		*P* value	*.002*		<.001	—				
		Robust SE	*0.03*		0.001	—				
	**Interaction effects added**									
		Coefficient	–0.0093		–0.0038	–*0.0044*				
		*P* value	.80		<.001	*.009*				
		Robust SE	0.04		0.001	*0.002*				
**Group 2: Low frequency or low comorbidity**										1,292,334
	**Direct effect**									
		Coefficient	–*0.0483*		–0.0082	—				
		*P* value	*<.001*		<.001	—				
		Robust SE	*0.01*		0.000	—				
	**Interaction effects added**									
		Coefficient	–0.0000		–0.0076	–*0.0025*				
		*P* value	.999		<.001	*<.001*				
		Robust SE	0.02		0.000	*0.001*				
**Group 3: High internet coverage**										851,569
	**Direct effect**									
		Coefficient	–*0.0500*		–0.0105	—				
		*P* value	*.001*		<.001	—				
		Robust SE	*0.02*		0.000	—				
	**Interaction effects added**									
		Coefficient	0.0161		–0.0095	–*0.0039*				
		*P* value	.42		<.001	*<.001*				
		Robust SE	0.020		0.000	*0.001*				
**Group 4: Low internet coverage**										*749,130*
	**Direct effect**									
		Coefficient	*0.0095*		–0.0031	—				
		*P* value	*.62*		<.001	—				
		Robust SE	*0.02*		0.001	—				
	**Interaction effects added**									
		Coefficient	0.0705		–0.0024	–*0.0027*				
		*P* value	.008		<.001	*.004*				
		Robust SE	0.03		0.001	*0.001*				

^a^Robust SEs clustered by patients.

^b^Constant is included in each of the models.

^c^Control variables including community-level, hospital-level, patient-level, and admission-level are included in the models.

^d^Year and hospital fixed effects are included in the models.

^e^Italicized values represent [insert reason].

^f^Not applicable.

From Table 4, we found that telehealth adoption has a larger impact on groups 1 and 3. Thus, following these findings, in our next set of post hoc tests, we focus on these 2 patient groups and study the impact of telehealth adoption among patients with high versus low virtualization potential diseases, using a classification based on the degree of process virtualization [26]—where physical interactions between people and objects are replaced by virtual interactions. Telehealth is likely more effective among patients with diseases with high virtualization potential (DHVP) compared with those with diseases with low virtualization potential (DLVP) [26]. Based on the classification suggested by Ayabakan et al [26], we categorized patients into 2 groups—DHVP and DLVP. DHVP include endocrine, nutritional, and metabolic diseases, mental illnesses, diseases of the skin and subcutaneous tissue, and musculoskeletal system diseases. All other disease categories were classified as DLVP. Our results, as shown in Table 5, indicate that the direct impact of telehealth adoption is more effective in reducing LOS for patients with DHVP compared with those with DLVP. These findings underscore how the effectiveness of telehealth in reducing LOS is influenced by disease type.

**Table 5:** Effect of telehealth adoption and drive time to hospital on length of stay by disease virtualization potential^a-d^.

Group	Direct effect of telehealth	Robust SE	*P* value	Observations, n
Group 5: High frequency, high comorbidity, and DHVP^e^	*–0.1736^f^*	0.03	.02	39,361
Group 6: High frequency, high comorbidity, and DLVP^f^	*–0.0731*	0.03	.01	269,004
Group 7: High internet coverage and DHVP	*–0.0825*	0.04	.04	136,252
Group 8: High internet coverage and DLVP	*–0.0491*	0.02	.001	715,317

^a^Robust SEs clustered by patients.

^b^Constant is included in each of the models.

^c^Control variables including community-level, hospital-level, patient-level, and admission-level are included in the models.

^d^Year and hospital fixed effects are included in the models.

^e^DHVP: diseases with high virtualization potential.

^f^Italicized values represent [insert reason].

^g^DLVP: diseases with low virtualization potential.

## Discussion

### Principal Findings

Overall, this study is among the first to comprehensively examine how telehealth mitigates health disparities associated with travel time to hospital for patients with recurrent admissions using a large, inpatient, longitudinal dataset in the United States. We make 2 main contributions. First, our study contributes to the health disparity literature by identifying the important role of telehealth in mitigating health disparities generated by distance barriers for patients with recurrent admissions. Previous literature pointed out that distance barriers are associated with decreased health outcomes [6,7] and called for studies to identify evidence for interventions that mitigate these adverse effects [11,51]. Our study answers this call and finds evidence that telehealth plays a significant role in bridging health disparities generated by distance barriers. Our results contribute to the literature by providing further evidence of the direct effect of drive distance on LOS, showing that the relationship is statistically significant and negative. One possible explanation for this phenomenon is that longer drive times might encourage using telehealth to better discharge planning and care coordination after discharge to avoid waste of resources [52], which reduces the duration of hospital stays. Furthermore, our result indicates that telehealth could be particularly effective in reducing in-hospital LOS for patients who live farther away from hospitals, potentially leading to more efficient use of hospital resources and better patient outcomes.

Second, our study examined the effects of telehealth on in-hospital LOS for patients with 2 or more admissions and various health conditions and diagnoses, thereby providing an in-depth understanding of the effects of telehealth. The result shows that telehealth adoption is not only associated with shortened in-hospital LOS but also reveals that the impact of telehealth is different for different patient groups. For example, telehealth has a larger impact on reducing in-hospital LOS for patients with complex health needs, needing frequent health services, and having DHVP. This may be due to telehealth facilitating better outpatient services [22,23] and medication management [53]. Such effects may be stronger for patients who frequently engage with health care systems and have complex health needs, potentially reducing their need for prolonged LOS further. Our results indicate telehealth could be effectively integrated into the care plans for those patient groups. Furthermore, the literature on how internet coverage influences the effects of telehealth on health outcomes is relatively sparse. Our study addresses this gap and finds that telehealth reduces in-hospital LOS for patients in high internet coverage areas and has no significant impact on those in low coverage areas. For patients living in high internet coverage areas, telehealth adoption is more effective for patients with DHVP. Future research should explore the underlying mechanisms of these findings.

**Figure 4:**
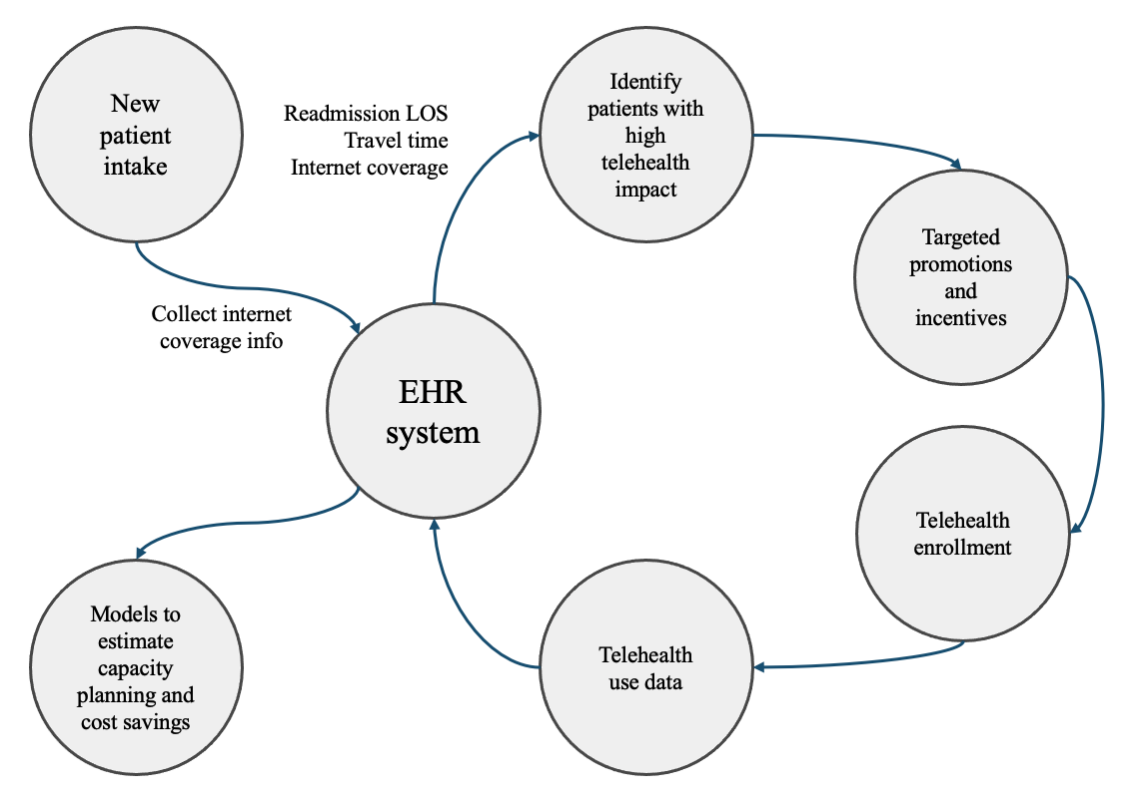
Proposed process changes and software implementation framework. EHR: electronic health record; LOS: length of stay.

The research also has practical implications. First, it informs health care practitioners and hospitals that telehealth can benefit patients who require regular monitoring and intervention and need more resources, such as patients with frequent care use and many comorbidities. Second, this research offers policy makers evidence that telehealth can mitigate certain health disparities caused by distance barriers for patients with recurrent admissions. Third, our results highlight the importance of patients living in areas with high internet coverage in order to fully reap the benefits of telehealth. This is particularly important for rural residents, where internet services are limited. Several independent nonprofit research groups have repeatedly called for extending broadband access in rural communities to enable telehealth access [50,54,55]. Furthermore, the Federal Communications Commission has stated that broadband access is a “super determinant of health” [48], underscoring the importance of internet access and its impact on public health. Thus, we recommend decision makers consider the internet coverage factor when making telehealth implementation–related decisions. Fourth, our findings have practical implications for hospitals and policy makers in resource-limited countries that are beginning to adopt telehealth. For example, our data indicate that telehealth is more effective in reducing LOS for patients with frequent hospital visits, multiple comorbidities, those living far from medical facilities, and those with patients with DHVP. Hospitals serving such demographics are likely to benefit substantially from telehealth. Finally, the results of our study might encourage actionable process changes and software implementations for the providers leading to improved patient care, cost savings, and better resource use. We present a framework for such implementations and process changes in Figure 4. This would be particularly beneficial for providers with high rehospitalization rates and longer average LOS and patients with relatively long travel times to the health care facility. Some of the software and process changes we propose based on our findings in this research are to (1) incorporate additional data such as travel time and internet coverage data during admissions and patient intake process to the electronic health record system; (2) develop a data extraction module based on patient readmission rates, LOS, travel time, and internet coverage and apply analytics to identify patients with potential high telehealth impact; (3) develop promotional and incentivizing strategies to encourage enrollment and use of telehealth and integrate such strategies with patient reach out and communication channels; (4) integrate telehealth enrollment and use data with the electronic health record system (if not already integrated); and (5) develop analytics and predictive models for improved capacity planning, use, and cost savings.

### Limitations and Future Research

Our study has a few limitations that also present opportunities for future research. First, our study is limited to data from the states of New York and Florida from 2012 to 2015, when telehealth services were not as pervasive as today. Future research could investigate whether the effects of telehealth observed in this study are consistent across other US states with more recent longitudinal data. Second, due to the broad definition of telehealth in our dataset, it is challenging to pinpoint which specific telehealth interventions contribute to the observed outcomes. Future research should focus on differentiating the effects of various telehealth modalities to better understand how they address health care delivery challenges, particularly those related to travel-time barriers. Finally, our analysis focuses on hospital-level telehealth adoption, which, while comprehensive, may be too broad to inform specific decisions for particular health care settings or hospital units that provide various treatments for specific diseases or conditions. Future studies could further examine the impact of telehealth at the departmental level or within particular medical contexts.

### Conclusions

Using a large panel dataset, this study contributes to the HIT and health disparity literature, providing strong empirical evidence that telehealth may mitigate certain health disparities generated by travel time–related barriers, with stronger effects for patients who visit hospitals more frequently, have a higher number of comorbidities, and live in high internet coverage area. Furthermore, it offers insights on guiding health care practitioners to allocate more resources to improve their telehealth access and service quality, especially for hospitals serving a large demographic of patients who live farther away from the hospital.

## Appendix

Multimedia Appendix 1 Checklist of items for the STROBE (Strengthening the Reporting of Observational Studies in Epidemiology) statement.

Multimedia Appendix 2 Health information technology items and coding.

Multimedia Appendix 3 Summary statistics for control variables and additional variables.

## Abbreviations

AHA: American Hospital Association
DHVP: diseases with high virtualization potential
DLVP: diseases with low virtualization potential
HCUP: Healthcare Cost and Utilization Project
HCUP-SID: Healthcare Cost and Utilization Project’s State Inpatient Datasets
HIT: health information technology
IT: information technology
LOS: length of stay
STROBE: Strengthening the Reporting of Observational Studies in Epidemiology

## Data Availability

Due to restricted usage imposed by the American Hospital Association (AHA) data use agreement and the Healthcare Cost and Utilization Project (HCUP) data use agreement, access to the datasets used in this study is limited. The dataset applied in this study can be accessed with permission from the AHA [33] and Agency for Healthcare Research and Quality [32].
