# Supplementary material for: Impact of Telehealth on Health Disparities Associated With Travel Time to Hospital for Patients With Recurrent Admissions: 4-Year Panel Data Analysis
Source: J Med Internet Res. 2024 Nov 25;26:e63661. doi: 10.2196/63661 (PMC11629038; doi:10.2196/63661)
Supplement: Multimedia Appendix 1 [file jmir_v26i1e63661_app1.docx]

## **Appendix 1. Checklist of Items for the STROBE Statement** [1]

Below, we present the checklist of the items in the STROBE statement.

|  | Item No | Recommendation | Checked  Result |
| --- | --- | --- | --- |
| **Title and abstract** | 1 | (*a*) Indicate the study’s design with a commonly used term in the title or the abstract | Check |
|  |  | (*b*) Provide in the abstract an informative and balanced summary of what was done and what was found | Check |
| Introduction | | |  |
| Background/rationale | 2 | Explain the scientific background and rationale for the investigation being reported | Check |
| Objectives | 3 | State specific objectives, including any prespecified hypotheses | Check |
| Methods | | |  |
| Study design | 4 | Present key elements of study design early in the paper | Check |
| Setting | 5 | Describe the setting, locations, and relevant dates, including periods of recruitment, exposure, follow-up, and data collection | Check |
| Participants | 6 | *Cross-sectional study*—Give the eligibility criteria, and the sources and methods of selection of participants | Check |
| Variables | 7 | Clearly define all outcomes, exposures, predictors, potential confounders, and effect modifiers. Give diagnostic criteria, if applicable | Check |
| Data sources/ measurement | 8* | For each variable of interest, give sources of data and details of methods of assessment (measurement). Describe comparability of assessment methods if there is more than one group | Check |
| Bias | 9 | Describe any efforts to address potential sources of bias | Check |
| Study size | 10 | Explain how the study size was arrived at | Check |
| Quantitative variables | 11 | Explain how quantitative variables were handled in the analyses. If applicable, describe which groupings were chosen and why | Check |
| Statistical methods | 12 | (*a*) Describe all statistical methods, including those used to control for confounding | Check |
|  |  | (*b*) Describe any methods used to examine subgroups and interactions | Check |
|  |  | (*c*) Explain how missing data were addressed | Check |
|  |  | (*e*) Describe any sensitivity analyses | N/A |
| Results | | | |
| Participants | 13* | (a) Report numbers of individuals at each stage of study—eg numbers potentially eligible, examined for eligibility, confirmed eligible, included in the study, completing follow-up, and analyzed | Check |
|  |  | (b) Give reasons for non-participation at each stage | N/A |
|  |  | (c) Consider use of a flow diagram | Check |
| Descriptive data | 14* | (a) Give characteristics of study participants (eg demographic, clinical, social) and information on exposures and potential confounders | Check |
|  |  | (b) Indicate number of participants with missing data for each variable of interest | N/A |
| Outcome data | 15* | *Cross-sectional study—*Report numbers of outcome events or summary measures | Check |
| Main results | 16 | (*a*) Give unadjusted estimates and, if applicable, confounder-adjusted estimates and their precision (eg, 95% confidence interval). Make clear which confounders were adjusted for and why they were included | Check |
|  |  | (*b*) Report category boundaries when continuous variables were categorized | N/A |
|  |  | (*c*) If relevant, consider translating estimates of relative risk into absolute risk for a meaningful time period | N/A |
| Other analyses | 17 | Report other analyses done—e.g. analyses of subgroups and interactions, and sensitivity analyses | Check |
| Discussion | | | |
| Key results | 18 | Summarize key results with reference to study objectives | Check |
| Limitations | 19 | Discuss limitations of the study, taking into account sources of potential bias or imprecision. Discuss both direction and magnitude of any potential bias | Check |
| Interpretation | 20 | Give a cautious overall interpretation of results considering objectives, limitations, multiplicity of analyses, results from similar studies, and other relevant evidence | Check |
| Generalizability | 21 | Discuss the generalizability (external validity) of the study results | Check |
| Other information | | | |
| Funding | 22 | Give the source of funding and the role of the funders for the present study and, if applicable, for the original study on which the present article is based | Check |

Reference:

1. Von Elm E, Altman DG, Egger M, Pocock SJ, Gøtzsche PC, Vandenbroucke JP. The Strengthening the Reporting of Observational Studies in Epidemiology (STROBE) statement: guidelines for reporting observational studies. The lancet. 2007;370(9596):1453-7.
