# Supplementary material for: Impact of Telehealth on Health Disparities Associated With Travel Time to Hospital for Patients With Recurrent Admissions: 4-Year Panel Data Analysis
Source: J Med Internet Res. 2024 Nov 25;26:e63661. doi: 10.2196/63661 (PMC11629038; doi:10.2196/63661)
Supplement: Multimedia Appendix 2 [file jmir_v26i1e63661_app2.docx]

## **Appendix 2.** **Health Information Technology Items and Coding**

We coded HIE implementation as a categorical variable, where 1 indicates telehealth is fully implemented across all units and 0 otherwise. We constructed HIT functions, including CDSS, CPOE, ECD, and RV, by counting the number of technology items fully implemented at a hospital. The detailed items for each function are listed below:

Clinical decision support system (CDSS):

1. Clinical guidelines (e.g. Beta blockers post‐MI, ASA in CAD)
2. Clinical reminders (e.g. pneumovax)
3. Drug allergy alerts
4. Drug‐drug interaction alerts
5. Drug‐lab interaction alerts
6. Drug dosing support (e.g. renal dose guidance)

Computerized provider order entry (CPOE):

1. Laboratory tests
2. Radiology tests
3. Medications
4. Consultation requests
5. Nursing orders

Electronic Clinical Documentation (ECD):

1. Patient demographics
2. Physician notes
3. Nursing notes
4. Problem lists
5. Medication lists
6. Discharge summaries
7. Advanced directives

Results viewing (RV):

Laboratory reports

Radiology reports

Radiology images

Diagnostic test results (e.g. EKG report, Echo report)

Diagnostic test images (e.g. EKG tracing)

Consultant reports
