# Supplementary material for: Impact of Telehealth on Health Disparities Associated With Travel Time to Hospital for Patients With Recurrent Admissions: 4-Year Panel Data Analysis
Source: J Med Internet Res. 2024 Nov 25;26:e63661. doi: 10.2196/63661 (PMC11629038; doi:10.2196/63661)
Supplement: Multimedia Appendix 3 [file jmir_v26i1e63661_app3.docx]

## **Appendix 3. Summary Statistics for Control Variables and Additional Variables**

Below, we present the summary statistics of the main dependent and independent variables, control variables, and the additional variables applied in the post hoc analyses.

Table S1. Summary Statistics for Control Variables and Additional Variables^a^

| **Variable name** | **Mean** | **std.** | **Min** | **Max** |
| --- | --- | --- | --- | --- |
| Hospital Characteristic |  |  |  |  |
| No. of beds | 824.998 | 715.382 | 40 | 2654 |
| For profit^†^ | 0.055 | 0.228 | 0 | 1 |
| Not for profit^†^ | 0.829 | 0.377 | 0 | 1 |
| In a Health System^†^ | 0.704 | 0.457 | 0 | 1 |
| Market Share | 0.138 | 0.105 | 0.048 | 1 |
| Other HITs |  |  |  |  |
| CDSS | 4.636 | 1.915 | 0 | 6 |
| CPOE | 3.180 | 2.290 | 0 | 5 |
| RV | 5.391 | 0.972 | 0 | 6 |
| ECD | 5.262 | 1.692 | 0 | 7 |
| HIE^†^ | 0.614 | 0.487 | 0 | 1 |
| Patient Characteristic |  |  |  |  |
| Age < 45^†^ | 0.122 | 0.327 | 0 | 1 |
| 45 <=Age < 65^†^ | 0.305 | 0.460 | 0 | 1 |
| 65 <=Age < 75^†^ | 0.211 | 0.408 | 0 | 1 |
| 75 <=Age <85^†^ | 0.214 | 0.410 | 0 | 1 |
| Age >=85^†^ | 0.150 | 0.357 | 0 | 1 |
| Female^†^ | 0.528 | 0.499 | 0 | 1 |
| White^†^ | 0.620 | 0.485 | 0 | 1 |
| Black^†^ | 0.162 | 0.368 | 0 | 1 |
| Hispanic^†^ | 0.140 | 0.347 | 0 | 1 |
| Asian/Pacific Islander^†^ | 0.016 | 0.125 | 0 | 1 |
| Native American^†^ | 0.002 | 0.044 | 0 | 1 |
| Race: Other^†^ | 0.059 | 0.236 | 0 | 1 |
| Patient Admission and Clinical Information |  |  |  |  |
| Medicare^†^ | 0.642 | 0.479 | 0 | 1 |
| Medicaid^†^ | 0.135 | 0.342 | 0 | 1 |
| Private insurance^†^ | 0.163 | 0.369 | 0 | 1 |
| No. of comorbidities | 3.237 | 1.907 | 0 | 14 |
| No. of chronic diagnoses | 6.513 | 3.204 | 0 | 25 |
| No. of diagnoses | 12.345 | 5.983 | 3 | 31 |
| No. of procedures | 1.906 | 2.471 | 0 | 31 |
| Transfer Body System^†^ | 0.439 | 0.496 | 0 | 1 |
| No. of Visits | 2.831 | 2.477 | 1 | 17 |
| Days in Between Visits | 108.244 | 183.767 | 1 | 1456 |
| Total Charge (in U.S. dollars) | 52297 | 60177 | 0 | 3791610 |
| Disease types |  |  |  |  |
| Infectious and parasitic disease^†^ | 0.069 | 0.254 | 0 | 1 |
| Neoplasms^†^ | 0.046 | 0.209 | 0 | 1 |
| Endocrine, nutritional, metabolic diseases, and immunity disorders^†^ | 0.049 | 0.215 | 0 | 1 |
| Diseases of blood and blood-forming organs^†^ | 0.023 | 0.149 | 0 | 1 |
| Mental disorders^†^ | 0.042 | 0.201 | 0 | 1 |
| Diseases of the nervous system and sense organs^†^ | 0.028 | 0.164 | 0 | 1 |
| Diseases of the circulatory system^†^ | 0.209 | 0.407 | 0 | 1 |
| Diseases of the respiratory system^†^ | 0.104 | 0.306 | 0 | 1 |
| Diseases of the digestive system^†^ | 0.113 | 0.317 | 0 | 1 |
| Diseases of the genitourinary System^†^ | 0.060 | 0.237 | 0 | 1 |
| Diseases of the skin and subcutaneous tissue^†^ | 0.024 | 0.153 | 0 | 1 |
| Diseases of the musculoskeletal system^†^ | 0.052 | 0.222 | 0 | 1 |
| Congenital anomalies^†^ | 0.001 | 0.033 | 0 | 1 |
| Symptoms, signs, and ill-defined conditions^†^ | 0.061 | 0.240 | 0 | 1 |
| Injury and poisoning^†^ | 0.092 | 0.289 | 0 | 1 |
| Factors influencing health status and contact with health services^†^ | 0.027 | 0.161 | 0 | 1 |
| Comorbidity Type |  |  |  |  |
| Acquired immune deficiency syndrome^†^ | 0.002 | 0.049 | 0 | 1 |
| Alcohol abuse^†^ | 0.049 | 0.215 | 0 | 1 |
| Deficiency anemias^†^ | 0.238 | 0.426 | 0 | 1 |
| Rheumatoid arthritis/collagen vascular diseases^†^ | 0.037 | 0.190 | 0 | 1 |
| Chronic blood loss anemia^†^ | 0.011 | 0.103 | 0 | 1 |
| Congestive heart failure^†^ | 0.124 | 0.330 | 0 | 1 |
| Chronic pulmonary disease^†^ | 0.236 | 0.425 | 0 | 1 |
| Coagulopathy^†^ | 0.065 | 0.246 | 0 | 1 |
| Depression^†^ | 0.132 | 0.338 | 0 | 1 |
| Uncomplicated diabetes^†^ | 0.263 | 0.441 | 0 | 1 |
| Diabetes with chronic complications^†^ | 0.071 | 0.256 | 0 | 1 |
| Drug abuse^†^ | 0.045 | 0.208 | 0 | 1 |
| Hypertension^†^ | 0.650 | 0.477 | 0 | 1 |
| Hypothyroidism^†^ | 0.149 | 0.356 | 0 | 1 |
| Liver disease^†^ | 0.044 | 0.206 | 0 | 1 |
| Lymphoma^†^ | 0.013 | 0.114 | 0 | 1 |
| Fluid and electrolyte disorders^†^ | 0.292 | 0.455 | 0 | 1 |
| Metastatic cancer^†^ | 0.031 | 0.173 | 0 | 1 |
| Other neurological disorders^†^ | 0.101 | 0.301 | 0 | 1 |
| Obesity^†^ | 0.138 | 0.345 | 0 | 1 |
| Paralysis^†^ | 0.038 | 0.190 | 0 | 1 |
| Peripheral vascular disorders^†^ | 0.087 | 0.282 | 0 | 1 |
| Psychoses^†^ | 0.054 | 0.226 | 0 | 1 |
| Pulmonary circulation disorders^†^ | 0.032 | 0.175 | 0 | 1 |
| Renal failure^†^ | 0.195 | 0.396 | 0 | 1 |
| Solid tumor without metastasis^†^ | 0.031 | 0.174 | 0 | 1 |
| Peptic ulcer disease excluding bleeding^†^ | 0.001 | 0.024 | 0 | 1 |
| Valvular disease^†^ | 0.060 | 0.238 | 0 | 1 |
| Weight loss^†^ | 0.048 | 0.215 | 0 | 1 |
| Community-level controls |  |  |  |  |
| Internet Coverage | 0.791 | 0.056 | 0.550 | 0.900 |
| Language Barrier (% Not Proficient in English) | 0.004 | 0.004 | 0.000 | 0.152 |

a: Dummy variables with two values: 0 and 1
